# Pulmonary arterial enlargement predicts long-term survival in COPD patients

**DOI:** 10.1371/journal.pone.0195640

**Published:** 2018-04-25

**Authors:** Juan P. de-Torres, Ana Ezponda, Ana B. Alcaide, Arantza Campo, Juan Berto, Jessica Gonzalez, Javier J. Zulueta, Ciro Casanova, Luisa Elena Rodriguez-Delgado, Bartolome R. Celli, Gorka Bastarrika

**Affiliations:** 1 Pulmonary Department, Clínica Universidad de Navarra, Pamplona, Spain; 2 Radiology Department, Clínica Universidad de Navarra, Pamplona, Spain; 3 Pulmonary Department, Tenerife, Spain; 4 Radiology Department, Hospital Ntra Sra de Candelaria, Tenerife, Spain; 5 Pulmonary Department, Brigham and Women Hospital, Boston, MA, United States of America; National and Kapodistrian University of Athens, GREECE

## Abstract

**Rationale:**

Pulmonary artery enlargement (PAE) is associated with exacerbations in Chronic Obstructive Pulmonary Disease (COPD) and with survival in moderate to severe patients. The potential role of PAE in survival prediction has not been compared with other clinical and physiological prognostic markers.

**Methods:**

In 188 patients with COPD, PA diameter was measured on a chest CT and the following clinical and physiological parameters registered: age, gender, smoking status, pack-years history, dyspnea, lung function, exercise capacity, Body Mass Index, BODE index and history of exacerbations in year prior to enrolment. Proportional Cox regression analysis determined the best predictor of all cause survival.

**Results:**

During 83 months (±42), 43 patients died. Age, pack-years history, smoking status, BMI, FEV1%, six minute walking distance, Modified Medical Research Council dyspnea scale, BODE index, exacerbation rate prior to enrollment, PA diameter and PAE (diameter≥30mm) were associated with survival. In the multivariable analysis, age (HR: 1.08; 95%CI: 1.03–1.12, p<0.001) and PAE (HR: 2.78; 95%CI: 1.35–5.75, p = 0.006) were the most powerful parameters associated with all-cause mortality.

**Conclusions:**

In this prospective observational study of COPD patients with mild to moderate airflow limitation, PAE was the best predictor of long-term survival along with age.

## Introduction

Chronic Obstructive Pulmonary Disease (COPD) is a highly prevalent disease and one of the leading causes of death worldwide [1]. Several noninvasive tools have been described to evaluate COPD survival: 1. Physiological, including degree of airway obstruction [2] or lung hyperinflation [3] (Inspiratory to Total Lung Capacity ratio: IC/TLC); 2. Clinical, such as degree of dyspnea measured by the modified Medical Research Council (MMRC) scale [4], exercise capacity evaluated by the six minute walking distance (6MWD) [5], nutritional status measure by body mass index (BMI) or free fat mass index (FFMI) [6], and presence of exacerbations [7]; 3. Composite indexes, such as the BODE index [8] (Body mass index, Obstruction, Dyspnea and Exercise capacity) or the ADO index [9] (Age, Dyspnea and Obstruction) or 4. Radiological, such as the presence of emphysema [10], bronchiectasis [11] or Pulmonary Artery Enlargement (PAE) [12].

Pulmonary hypertension (PH) is a progressive, life threating condition defined by the presence of a mean pulmonary artery pressure ≥25 mm Hg [13]. It is usually associated to severe degree of airway obstruction with a prevalence of 25–35% in this group of patients, but it has also been described as frequent as 5–7% in patients with mild to moderate disease [14]. The gold standard for its diagnosis is the invasive measurement of the mean Pulmonary Artery Pressure (mPAP) by right heart catheterization [13]. Several noninvasive tools have been described for the screening for PH, including Transthoracic Doppler Ultrasound measurement of the systolic PAP from the peak tricuspid regurgitation jet or the detection of an enlarged PA in a chest CT defined by a main PA diameter >30mm or a main PA to ascending Aorta ratio>1 [15].

An enlarged PA, usually evaluated with an PA/Aorta ratio>1, has been associated with the number of exacerbations [16], exercise capacity (6MWD distance) [17] and just recently, with all-cause mortality but only in patients with moderate to severe disease [12].

The potential role of an enlarged PA (evaluated by a diameter≥30mm) as a prognostic marker of all degrees of airway limitation (AL) severity in COPD patients has not been compared with other well-consolidated prognostic markers of the disease.

We therefore prospectively recruited COPD patients that attend our pulmonary clinic in order to compare the prognostic value of an enlarged PA (diameter >30mm) detected in a chest CT against other well-established prognostic markers of COPD survival.

## Methodology

This a prospective observational study of a COPD cohort attending a Pulmonary Clinic (ClinicalTrials.gov Identifier: NCT01122758).

Participants were ever smokers of at least 10 pack-years with previous spirometric diagnosis of COPD (post-bronchodilator FEV1/FVC ratio of less than 0.7 after the inhalation of 400μg of Salbutamol). All patients had to be clinically stable (no exacerbations) for 8 weeks and receiving optimal therapy [2]. Exclusion criteria were uncontrolled co-morbidities such as malignancy or other confounding diseases: severe congestive heart failure, obliterative bronchiolitis, or diffuse panbronchiolitis. Our Institution`s ethics committee (Clínica Universidad de Navarra) approved the study (IRB approval n° 28/2012) and all patients signed the informed consent form.

### Clinical variables

At recruitment, trained personnel obtained sociodemographic information and smoking history, including age, gender, current smoking status, and intensity of exposure. Using these data, we calculated the total smoking exposure, in pack-years, of each participant.

Lung function and DLCO were measured according to the ATS/ERS guidelines [18]. The 6-min walking distance (6MWD) was performed according to ATS guidelines [19]. Patients`dyspnea was evaluated with the Modified Medical Research Council (MMRC) scale [20]. Body Mass Index (BMI) was calculated in kg/m^2^. FEV_1_% of predicted values, BMI, 6MWD and MMRC values were integrated into the BODE index [8] as previously described and also classified in quartiles. Degree of AL was classified according to the Global Initiative for Chronic Obstructive Lung Disease (GOLD) criteria [2] into four groups: stage 1 (mild, post-bronchodilator FEV1≥80% predicted), stage 2 (moderate, post-bronchodilator FEV1 ≥50% to <80% predicted), stage 3 (severe, post-bronchodilator FEV1 <50% to ≥30% predicted), and stage 4 (very severe, post-bronchodilator FEV1 <30% predicted).

Exacerbations were defined by worsening of respiratory symptoms beyond normal daily variations that required the use of antibiotics, steroids or both, medical consultation or admission to hospital [2].

Survival was determined by direct follow-up with participants and/or their family.

### CT image acquisition and reconstruction protocol

All individuals were imaged on a multidetector CT system (Somatom Definition and Somatom Sensation 64, Siemens Healthcare, Forchheim, Germany) at time zero of the follow up time. Most individuals (n = 143) underwent low dose chest CT (LDCT) examination with the following parameters: 120 kV, 40 mAs, 32×0.6 mm detector collimation, pitch 1. Images were reconstructed with 5 mm and 1 mm slice thickness using soft tissue (B31f) and high-resolution (B60f) reconstruction algorithms to evaluate the mediastinum and lung parenchyma, respectively. Intravenous contrast was administrated in 46 patients.

#### Assessment of rmphysema on CT

All images were read by two chest radiologists (A.E. and G.B.) for visual assessment of the presence of emphysema, using validated criteria [21]. In brief, the extent of emphysema was graded from 0 to 4, with a grade of 0 indicating no emphysema, and a grade of 4 indicating the presence of emphysema in 75% of the lung.

#### Measurement of pulmonary diameter

**Fig 1:**
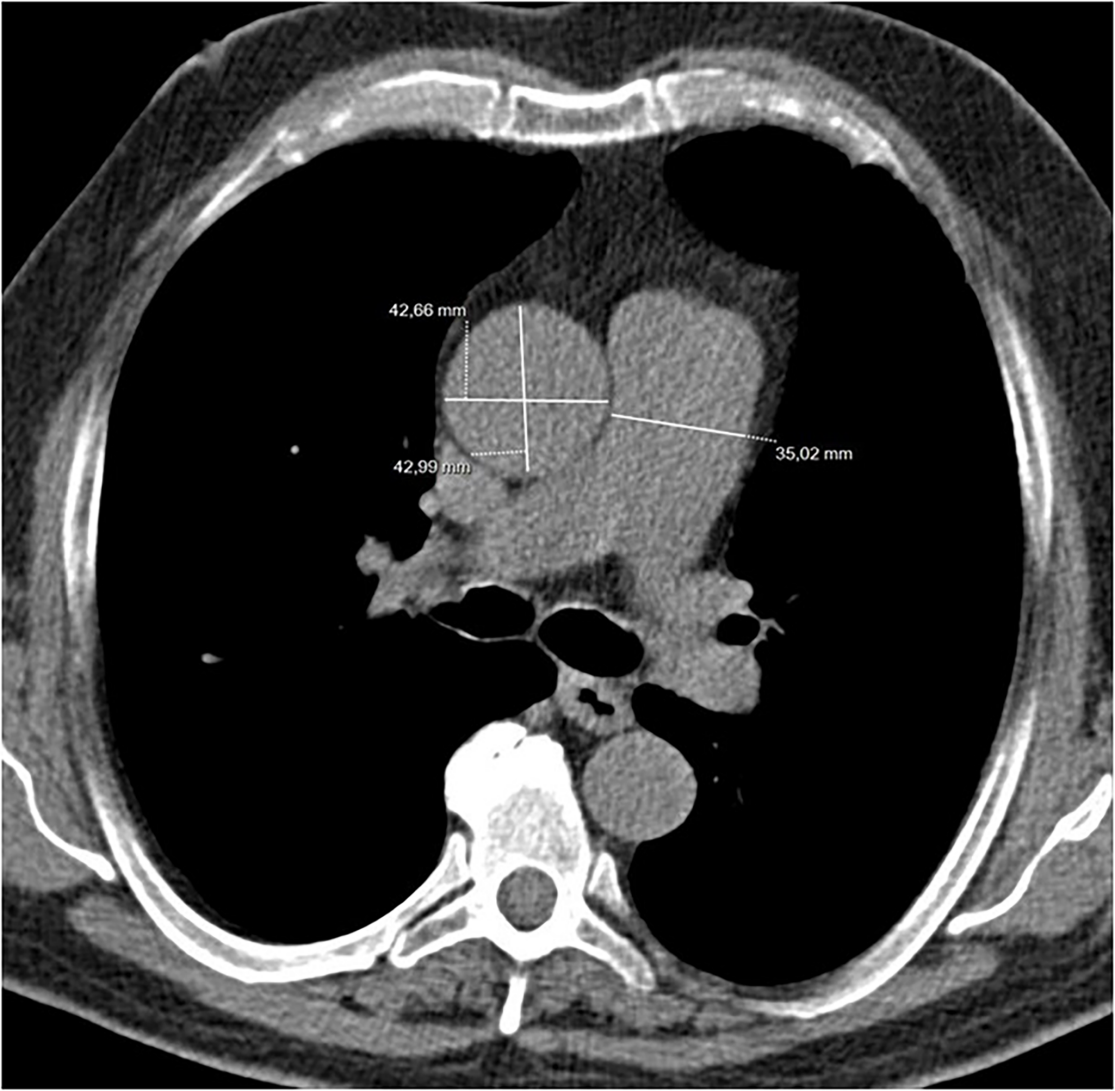
LDCT examination. Measurements of main Pulmonary Artery (PA) diameter and Ascending Aorta (AA) at the level of the bifurcation. The greatest diameter of the AA was used to calculate the PA/Aorta ratio.

Two thoracic radiologists blinded to patients’ clinical data measured the main pulmonary artery (PA) diameter at the level of its bifurcation on transverse CT images (Fig 1)

The greatest diameter of the ascending aorta (AA) was also measured at the same level. A cut off value of ≥30 mm was employed for distinguishing between patients with and without pulmonary hypertension. The ratio of the main PA diameter to the diameter of ascending aorta (PA/Aorta ratio) was also determined and a ratio >1 was considered an indirect marker of pulmonary hypertension, as previously described in COPD patients [22].

### Statistical analysis

Data was summarized as relative frequencies for categorical variables, mean (SD) for normally distributed variables. Pearson´s coefficient was calculated to explore the association between PA diameter and the prognostic variables.

A proportional Cox survival analysis first determined the independent association of each study parameter with all cause survival. Then, a multivariable analysis including all that had a statistical significant association in the univariable analysis determined the best predictor of all cause survival. Because some of the factors that were found to be independently associated with survival in the univariable analysis are part of the BODE index (BMI, FEV1%, MMRC and 6MWD), we only included this score representing all of them in the multivariable analysis, as this index showed to have the strongest prognostic power in previous studies. Significance level was established as a two-tailed p-Value ≤0.05. We used SPSS 22.0, Chicago, U.S.A. for the statistical analysis.

## Results

**Fig 2:**
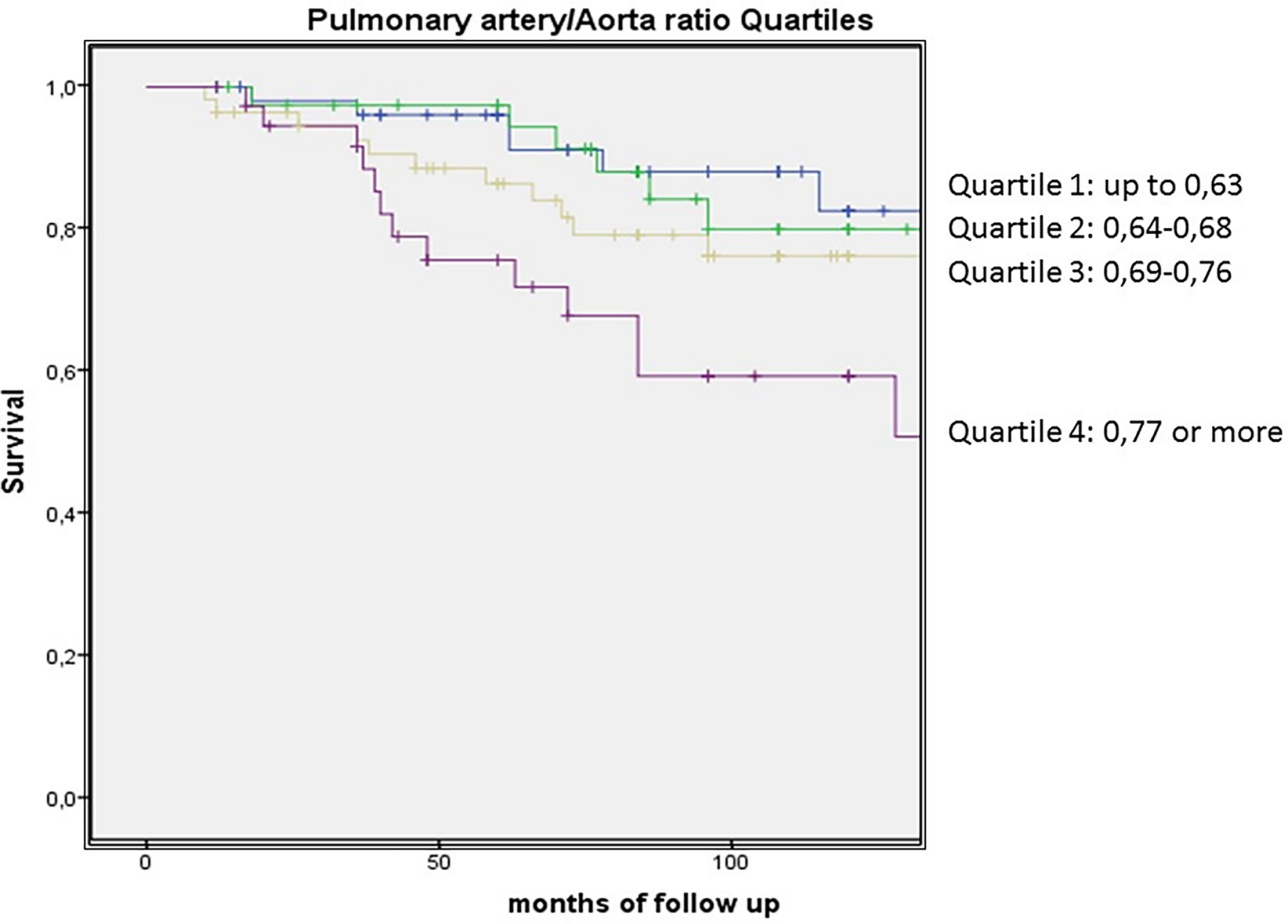
Flowchart showing the inclusion of patients for the final statistical analysis.

From an initial sample of 215 patients, we were able to obtain appropriate radiological information in 188 individuals: 23 had different acquisition technique and in 4, PA measurement were not able to be performed (Fig 2).

Table 1 shows the clinical, physiological and radiological characteristics of the participants.

**Table 1:** Clinical, radiological and physiological characteristics of the patients.

Patients characteristics	
n	188
Age (years)	65 ± 8
Follow up (months)	83 ± 42
Gender (%) male/female	82/18
Pack-year (units)	51 ± 27
Current Smoker (%) yes/no	35/65
FEV_1_/FVC (%)	55 ± 10
FEV_1_%	70 ± 20
FVC %	98 ± 20
TLC %	102 ± 15
DLCO	70 ± 20
MMRC 0-1-2-3-4 (%)	36-41-1-8-2
6MWD (m)	468 ± 112
BMI (Kg/m2)	26 ± 4
BODE Quart 1–4 (%)	80/10/8/2
BODE	1.3 ± 1.8
Spirometric GOLD stages I-II-III-IV %	36, 48, 14, 2
Exacerbations in the year prior to enrollment n	0.7 ± 0.9
Pulmonary Artery diameter mm	24.7 ± 4.1
Pulmonary Artery diameter≥30mm n, %	22, 12%
Pulmonary Artery diameter≥30mm by GOLD I+II / III+IV %	10/23
Pulmonary Artery/Aorta Artery ratio	0.69 ± 0.11
Pulmonary Artery/Aorta Artery ratio >1 n, %	3, 0.01%
Pulmonary Artery/Aorta Artery ratio quartiles %	29-21-30-20
Emphysema severity (visual) 0-1-2-3%	46-24-20-10
Deaths n, %	43, 23%
Deaths by GOLD I+II / III+IV %	22/30

n = Number of participants; BMI = Body Mass Index; FEV_1_ = Forced Expiratory Volume in the first second; FVC = Forced Vital Capacity; TLC = Total Lung Capacity; MMRC = Modified Medical research Council; 6MWD = 6 Minute Walk Distance; BODE index: BMI, Obstruction, Dyspnea, Exercise capacity, X ± SD = means ± Standard Deviation

They were mainly men with mild to moderate COPD, followed for a mean time of almost 7 years. Most of them were just mildly symptomatic, slightly overweight with a good exercise capacity at the time of enrollment. They had a low BODE score and less than one exacerbation in the year prior to enrollment. Twenty two (12%) patients had a PA diameter ≥30mm and only 3 patients presented a PA/Aorta ratio>1. Fifty four percent of them had emphysema, 30% of them showing more than 50% of emphysema in their LDCT.

During the follow up time, 43 deaths occurred. As expected, patients with a greater degree of airway obstruction (22% vs. 30%) had higher mortality.

Table 2 shows the clinical, physiological and radiological characteristics of those patients with PAE (n = 22).

**Table 2:** Clinical, radiological and physiological characteristics of the patients diagnosed with and without PAE.

Patients characteristics		
	With PAE	Without PAE
n	22	166
Age (years)	69 ± 6	64±8*
Follow up (months)	62 ± 38	88±42*
Gender (%) male/female	77/23	83/17*
Body Surface Area	1.88±0.23	1.87±0.22
Pack-year (units)	65 ± 32	49±26
Current Smoker (%) yes/no	27/73	35/65*
FEV_1_/FVC (%)	53 ± 12	56±10
FEV_1_%	55 ± 20	73±19*
FVC %	80 ± 18	101±19*
TLC %	88 ± 21	105±13
DLCO	62 ± 22	71±20*
MMRC 0-1-2-3-4 (%)	14-50-14-18-4	42-37-12-5-4
6MWD (m)	374 ± 129	482±103*
BMI (Kg/m2)	28 ± 5	26±5
BODE	2.2 ± 2.3	1.1±1.7
Spirometric GOLD stages I-II-III-IV %	9, 59, 23, 9	40-46-12-2*
Exacerbations in the year prior to enrollment n	1.2 ± 1.2	0.6±0.8*
Pulmonary Artery diameter mm	32.9 ± 3.5	23.7±2.8*
Emphysema severity (visual) 0-1-2-3%	64-18-14-4	44-25-21-10
Deaths n, %	12, 55%	31, 18%*

n = Number of participants; BMI = Body Mass Index; FEV_1_ = Forced Expiratory Volume in the first second; FVC = Forced Vital Capacity; TLC = Total Lung Capacity; MMRC = Modified Medical research Council; 6MWD = 6 Minute Walk Distance; BODE index: BMI, Obstruction, Dyspnea, Exercise capacity, X ± SD = means ± Standard Deviation

* statistically significant

Those with PAE were older, had higher consumption of tobacco, more likely to be women, had a higher BMI, more severe GOLD stage and BODE index, with a lesser burden of emphysema, a lower 6MWD and a higher degree of dyspnea.

Table 3 shows the association of the explored variables with PA diameter.

**Table 3:** Association between pulmonary artery diameter and other studied variables.

Variables	Pearson coefficient	p value
Age	0.28	0.001
Pack-year	0.18	0.013
BMI	0.28	0.001
Exacerbations in the year prior to enrollment	0.16	0.027
FEV1%	-0.24	0.001
DLCO	-0.12	0.13
MMRC	0.18	0.01
6MWD	-0.39	0.001
BODE	0.20	0.006
Emphysema severity (visual)	-0.13	0.08

As previously described in the literature, an enlarged PA diameter was associated with 6MWD, BMI, MMRC, FEV_1_%, BODE and the number of exacerbations reported the year prior to enrollment. We did not find an association of this variable with pulmonary emphysema severity nor with DLCO values.

To select the best PA parameter that predicted survival in our cohort of patients, we firstly explored the association between PA enlargement (PAE) (previously defined as a diameter ≥30mm) and PA/Aorta ratio quartiles. Results showed a Polychoric correlation [23] of rho = 0.76; bootstrap bias corrected 95%CI: (0.58; 0.88). Then we explored with a Cox proportional analysis the independent association with survival, showing that both the PA/Aorta ratio quartiles and PAE were associated with survival (HR: 3.73; 95%CI: 1.43–9.74, p<0.001, p for trend 0.05 and HR: 4.52; 95%CI: 2.28–8.98, p<0.001). Comparing the highest PA/Aorta quartile vs the other PA/Aorta quartiles, the HR was 2.22; 95%CI: 1.16–4.24, p = 0.016. When we compared the predicted power of both measurements in a multivariable analysis, PAE was a stronger predictor of survival with a HR: 4.52; 95%CI: 2.28–8.98, p<0.001 than PA/Aorta ratio quartiles HR: 2.20; 95%CI 0.77–6.27, p = 0.13. Therefore, we used PAE as a predictor of survival in the final model.

Table 4 Panel A. shows the results of Cox proportional risk analysis exploring the association between the evaluated parameters and all-cause mortality.

**Table 4:** Panel A. Univariable analysis showing the independent association of each parameter with survival. Panel B. Multivariable analysis showing those that remained in the model.

**Panel A. Univariable analysis exploring factors that predict survival**		
**Variables**	**HR (95% IC)**	**p value**
Age (for each year)	1.09 (1.05–1.13)	0.001
Pack-year (for each pack-year)	1.00 (1.01–1.02)	0.06
BMI (index)	1.00 (0,94–1,07)	0.80
Gender (male as reference)	0.45 (0.18–1.16)	0.10
Current Smoker (yes vs. no)	2.12 (1.02–4.39)	0.04
FEV1% (for each %)	0.97 (0.96–0.99)	0.001
MMRC (for each point)	1.36 (1.07–1.70)	0.01
6MWD (for each m)	0.99 (0.98–0.99)	0.001
BODE (for each point)	1.19 (1.04–1.35)	0.007
Pulmonary Artery diameter (for each mm)	1.19 (1.12–1.27)	0.001
Pulmonary Artery diameter≥30mm (yes vs. no)	4.52 (2.28–8.98)	0.001
Pulmonary artery quartiles (reference Q1)		
Q2 vs Q1	0.94 (0.32–2.71)	0.90
Q3 vs Q1	1.26 (0.62–4.22)	0.32
Q4 vs Q1	5.36 (2.33–12.32)	0.001
Emphysema (yes vs no)	0.88 (0.48–1.63)	0.69
**Panel B. Multivariable analysis**		
**Variables**	**HR (95%IC)**	**p value**
Age (for each year)	1.08 (1.04–1.13)	0.001
Pulmonary Artery diameter≥30mm (yes vs no)	2.78 (1.35–5.75)	0.006

Variables included in the model: age, BSA, current smoker, BODE, Pulmonary Artery diameter>30mm

We first confirmed that well recognized survival predictors in COPD patients were also predictors of survival in our population: age, smoking status, MMRC, FEV_1_%, 6MWD and BODE index. PA diameter as a continuous variable and as a categorical variable using the cut-off associated with PH (≥30mm), was independently associated with all cause survival. Factors usually associated with survival in COPD patients such as BMI and emphysema presence were not associated with survival in our population.

Table 4 Panel B. shows the results of the multivariable Cox regression analysis for those variables statistically significant in the univariable analysis. Being older and with a PA diameter ≥30mm were the most powerful predictors of survival in our cohort, stronger than the BODE index which did not retained in the final model.

**Fig 3:**
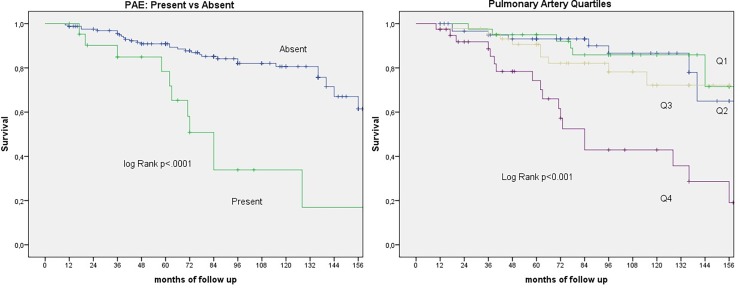
Kaplan Meier curves showing the cumulative survival for those with and without PAE and for pulmonary artery diameter quartiles.

Fig 3 shows the Kaplan Meier curves with the cumulative survival for those with and without PAE and for Pulmonary Artery diameter Quartiles.

## Discussion

The most important and novel finding from this mainly male COPD cohort, is that having a main PA diameter ≥30mm as measured on chest CT is a powerful and independent predictor of survival, providing greater prognostic information than well-established predictors of survival such as the BODE index.

The Framingham Heart Study provided the largest population based cohort (n = 706) to define normal values for main PA size measurement in non-contrast chest CT [24]. In this “healthy” population based cohort (nonsmokers without hypertension, obesity, COPD, thromboembolic disease, diabetes mellitus, cardiovascular disease or heart surgery) the mean value for main PA was 25.1±2.8mm with a 90^th^ percentile for men at 28.9mm and for women at 26.9mm. A recent meta-analysis of 12 studies [25] including different types of patients, established a cut-off value of 29.5mm has a sensitivity of 87% and specificity of 89% for PH and therefore is usually recommended for PH screening acknowledging that even a normal diameter cannot exclude the presence of PH. The ratio between main PA to ascending Aorta diameters has also been postulated as an indirect measurement for the presence of PH. The same Framingham Heart Study also provided the normal values for this ratio in a population based cohort: 0.77±0.09mm with a 90^th^ percentile cut off value at 0.91mm. The literature suggests that this ratio is a better predictor of PA pressure [24].

The main goal of the present work was to compare the prognostic power of PAE evaluation with existing clinical and physiological tools for survival and not to diagnose PH in COPD patients. Patients´ mean PA diameter was 24.7mm, falling within what is considered normal for a mainly male population, but 22 patients (12%) with a main PA diameter ≥30mm. Only 7 patients (3.7%) have what is considered an abnormal PA/Aorta ratio (>0.91). In our patients, PAE (≥30mm) correlated well with the PA/Aorta, but was a better predictor of survival than PA/Aorta ratio quartiles. Having a main PA diameter≥30mm and an older age were the best predictors of all of the tested variables. Terzikhan et al.[17] from the population based Rotterdam Study has recently provided information regarding the relationship between PAE and all-cause mortality in COPD patients. They studied a large sample of individuals from the general population (n = 2197) and as expected, 10% of them (222 patients) had pre-bronchodilator AL or COPD. They found that PA/Aorta ratio quartiles (largest vs shortest) were predictive of all-cause mortality but only in those with moderate to severe AO. There are several differences between that study and the present one. Firstly, Terzikhan´s is a population based study, where the diagnosis was made with pre bronchodilator spirometry where up to 30% of these patients may not have COPD in post bronchodilation according with previous data [26], while this study was conducted in a well characterized population with clinical COPD. Secondly and most important, Terzikhan et al. did not compare the predictive power of PAE with other well established prognostic tools of the disease. A PA diameter≥30mm was a stronger predictor of survival even than the multimensional BODE index, that has been consistently shown to be the best predictor of all-cause mortality in COPD patients [27]. This implies that independently of the degree of AL of our patients or in which BODE quartile they are, knowing the size of their main PA could further help physicians predict their survival. In clinical practice, most of our COPD patients usually get a LDCT for several reasons: for early detection of lung cancer [28], to determine the presence and distribution of emphysema to guide a potential lung volume reduction procedure [29], to explore the presence of bronchiectasis [11], to determine the presence of coronary calcium [30] and now we are also providing novel information to help in determining their survival.

Shin et al.[22] also explored the relationship between PA diameter and mortality in patients with COPD. In a sample of 65 patients from an Advanced Lung Disease clinic for potential lung transplantation or volume reduction surgery, these investigators found that those with PH proven on right heart catheterization had a larger mean PA diameter than those without PH: 34.4mm vs 29.1mm, p = 0.0003. They also found that those with a PA/Aorta ratio >1 had a reduced mortality although in a highly selected population of severe COPD patients.

As expected, PA diameter significantly correlated with age, pack year history and BMI. As has been shown previously it also correlated (although weakly) with the history of exacerbations the year prior to enrollment [15]. All components of the BODE index and the index itself statistically correlated well with PA diameter. The strongest correlation was found with 6MWD (r = -0.39, p<0.001) implying that even in this middle age mild to moderate COPD population the presence of an enlarged PA negatively impact on exercise capacity.

Potential explanations for an enlarged PA in a COPD are many [13]. Chronic hypoxemia secondary to lung parenchyma destruction due to emphysema is the most frequent one, even more in these patients with up to 30% of them have grade 2–3 (moderate to severe) visual emphysema that could have not only intermittent hypoxemia but also pulmonary vessels destruction. Both emphysema severity and DLCO were not associated with main PA diameter in our cohort. Peinado et al. showed in transplanted lungs from severe COPD patients the association of emphysema and pulmonary vascular remodeling [31]. Co-existence of Obstructive Sleep Apnea (OSA) could be as highly prevalent as 30% in COPD patients [32] and a frequent cause of PH [33]. Vascular remodeling and endothelial dysfunction are a common feature even in patients with mild to moderate disease [13] as a consequence of chronic intermittent hypoxia and smoking.

There are several limitations in the present work. Firstly, these findings come from a relatively small sample of COPD patients usually followed at a pulmonary clinic and therefore could not be extrapolated to other types of COPD patients. Secondly, these findings should be restricted to male COPD patients since most of the participants were men. A similar study should be conducted in female COPD patients to confirm our findings. Thirdly, unfortunately an important and frequent determinant of PH in COPD patients such as OSA has not been explored. Nevertheless, independently of the presence or not of OSA, the detection of an enlarged PA still could predict patient`s survival. Fourthly, although BSA was included in the multivariable analysis to correct PA size for each patient characteristic, this model included PAE as a dichotomous ordinal variable and not the PA size a continuous variable that could be more appropriate. Lastly this is a single centre study and replication is required in a multicenter study.

In conclusion, in a population of mainly male COPD with mild to moderate AL and followed in our pulmonary clinic, the presence of an enlarged (diameter≥30mm) PA was the strongest predictor of all cause survival along with age, even stronger than most of the clinical and physiologic prognostic parameters for the disease, including the well-validated multidimensional BODE index.

## Supporting information

S1 File “EPOC-TAC PlosOne.sav”. (SAV)

## Abbreviations

COPD: Chronic Obstructive Pulmonary Disease
MRC: modified Medical Research Council scale
6MWD: six minute walking distance
BMI: body mass index
FFMI: free fat mass index
PH: pulmonary hypertension
PAE: Pulmonary Artery Enlargement
mPAP: Mean Pulmonary Artery Pressure
AL: airway limitation
ATS: American Thoracic Society
ERS: European Respiratory Society
GOLD: Global Initiative for Chronic Obstructive Lung Disease
FEV1: Forced expiratory volume in the first second
AA: ascending aorta
OSA: Obstructive Sleep Apnea
